# P173: Effectiveness of a hospital-wide educational programme for infection control to reduce the rate of health-care associated infections and related sepsis (alerts) – first results

**DOI:** 10.1186/2047-2994-2-S1-P173

**Published:** 2013-06-20

**Authors:** S Hagel, K Ludewig, J Frosinski, R Hutagalung, P Gastmeier, S Harbarth, FM Brunkhorst

**Affiliations:** 1 Center for Infectious Diseases and Infection Control, Jena University Hospital, Jena, Germany; 2 Center for Sepsis Control and Care, Jena University Hospital, Jena, Germany; 3 Institute of Hygiene and Environmental Medicine, University Medicine, Charité, Berlin, Germany; 4 Infection Control Programme, Geneva University Hospitals, Geneva, Switzerland; 5 Paul Martini Sepsis Research Group, Jena University Hospital, Jena, Germany

## Objectives

The overarching objective of this clinical trial is to demonstrate the feasibility of an institutional programme to reduce the burden of HAIs and related sepsis of at least 20%, without targeting only specific pathogens or hospital wards.

## Methods

Prospective, quasi-experimental study covering all acute care units (27 general wards, 4 ICUs, overall 819 beds) at JUH. Surveillance for HAIs is performed by computerized antibiotic monitoring in patients with risk factors for HAIs (i.e. catheters, operations) on a daily basis. Following the 1^st^ surveillance period a multifaceted, pragmatic infection control programme, aimed at proper hand hygiene and bundles for the prevention of the four most common HAIs will be implemented. Subsequently, 2^nd^surveillance phase lasting 18 months will be conducted to measure the effect of the infection control programme, starting in May 2013.

## Results

Interim results for the first surveillance period (09/2011 to 08/2012) are presented. During this period 38.098 patients were admitted to the participating departments. According to CDC definitions we identified 1727 HAIs, resulting in an overall incidence of 4.5%. Based on clinical evaluation only, irrespective of the CDC definitions, an additional 868 HAIs were detected (overall HAI rate, 6.8% [n =2595]). A substantial proportion of patients had severe sepsis/septic shock due their HAI (LRTI, n=278 (37%); SSI, n=114 (25%); CLABSI, n=124 (33%); UTI, n=45 (8%); other, n=87 (22%).

## Conclusion

Our numbers confirm the current estimates of the incidence of HAIs in Germany. Furthermore, a high percentage of HAIs results in severe sepsis/septic shock, requiring ICU treatment.

## Disclosure of interest

None declared

